# The association between entrapment and depression among migrant workers in China: a social rank theory based study

**DOI:** 10.1186/s12888-021-03665-6

**Published:** 2022-01-06

**Authors:** Rusi Long, Hui Chen, Tian Hu, Yaqi Chen, Bolin Cao, Rongxi Wang, Fan Hu, Chen Xu, Xiaoyue Yu, Yujie Liu, Shangbin Liu, Kechun Zhang, Huachun Zou, Zixin Wang, Wenjie Xue, Yong Cai

**Affiliations:** 1 School of Public Health, Shanghai Jiao Tong University School of Medicine, Shanghai, China; 2 Shenzhen Longhua District Center for Disease Control and Prevention, Shenzhen, China; 3 School of Media and Communication, Shenzhen University, Shenzhen, China; 4 School of Public Health (Shenzhen), Sun Yat-sen University, Shenzhen, China; 5 Kirby Institute, University of New South Wales, Sydney, Australia; 6 JC School of Public Health and Primary Care, Faculty of Medicine, The Chinese University of Hong Kong, Hong Kong, China; 7 Ban Song Yuan Road Community Health Service Centre, Shanghai, China

**Keywords:** Migrant workers, Entrapment, Depressive symptoms, Social rank theory

## Abstract

**Background:**

Migrant workers are a group susceptible for depression evolution due to occupational maladaptive triggers. The social rank theory illustrates the pathology process from defensive adaptation to depression, pointing out the early prevention of depression by discovering entrapment. This study aims to reveal the relationship between migrant workers’ entrapment and depressive symptoms.

**Methods:**

A total of 1805 migrant workers in Shenzhen were recruited by stratified multi-stage sampling. Sample’s demographic, behavioral and psychosocial characteristics were described and analyzed to reveal the relationship between entrapment and depressive symptoms. The Receiver Operator Characteristic was performed to find the optimal cut-off point of Entrapment Scale for predicting depressive symptoms.

**Results:**

In the binary logistic regression of sociodemographic variables, migrant workers who were married (univariate odds ratio (ORu) = 0.69, 95% Confidence Interval (CI) = 0.56–0.84), owned 1 or 2 children (ORu = 0.71, 95% CI = 0.58–0.86), had been working over 10 years (ORu = 0.71, 95% CI = 0.54–0.95), earned > 4999 yuan per month (ORu = 0.68, 95% CI = 0.47–0.99; multivariate odds ratio (ORm) = 0.57, 95% CI = 0.38–0.87) or with low risks of alcohol use disorders (ORu = 0.51, 95% CI = 0.34–0.75) had lower risks of depressive symptoms. After adjusted the aforementioned significant sociodemographic variables, migrant workers with severer entrapment were more likely to have depressive symptoms (adjusted odds ratio (ORa) = 1.13, 95% CI = 1.12–1.15). Besides, the study proved the reliability and validity of the Chinese version Entrapment Scale, preferring a two-dimensional structure, and 11 was the optimal cut-off value of this scale for predicting depressive symptoms among migrant workers.

**Conclusions:**

This result indicates the potential value of entrapment according to the social rank theory on facilitating early prevention of migrant works’ depression and the application value of Entrapment Scale for effectively measuring mental status among migrant workers.

**Supplementary Information:**

The online version contains supplementary material available at 10.1186/s12888-021-03665-6.

## Introduction

Nowadays, occupational migration for economic reason has become a global phenomenon, greatly promoting the society production development [1]. In China, the economic reform leads the eastern coastal urban regions of China become the front line of industry as well as the major destinations for occupational migrants. According to the document publicized by government, Shenzhen, the one of the earliest Special Economic Zone of reform and opening, hosts more than 3 million manufactory workers in 2018 which accounts 28% of its residents [2]. Migrant Workers (MWs), migrating temporarily or seasonally to other regions for occupational opportunities, are commonly believed as low social ranked because they usually engage in unstable and unskilled works with economic vulnerability [3]. The negative sociocultural, economic and psychological change which includes separation from family and familiar social surroundings [4], exclusion from social welfare service [5], discrimination of mobile status [6] and poor living conditions [7, 8], expose MWs in a great risk of experiencing adverse psychological consequences [9–11]. What’s more, the emerging infectious diseases further deteriorate the health inequality as it increases unemployment risk, fuels negative emotion and limits medical resources [12].

Occupational related factor has become one of the most important maladaptive triggers for depression evolution [13]. Current available findings implied that compared with non-migrants, MWs among worldwide have a higher prevalence of depressive symptoms [14–17], particularly for female MWs [14, 17, 18]. MWs’ major depressive disorders would lead to several negative effects, such as disability [19], increasing the risks of unsafe sexual behavior [20, 21],decreasing the health-related quality of life [22] and so on, and hence cause a reduction of work productivity and economic growth. Thus, methods should be explored for discerning depression earlier.

The Social Rank Theory (SRT) endeavors to explain the evolutionary of depression through its fundamental symptoms—defeat and entrapment [23, 24], performing entrapment as an entry point to predict and prevent depression. It proposes that faced with unfavorable situation, such as unachievable goals or negative relationship, individuals who lack social power and whose attempts to change or challenge are inhibited will involuntarily yield adaptive responses by automatic shutdown strategy to facilitate accepting current situation [25]. If the situation is unacceptable, unchangeable or inescapable that the adaptive defensive responses can become maladaptive and eventually result in depression [26, 27]. Entrapment, a key maladaptive defensive response to provoke depression in SRT, is described as a common scenario under the adverse circumstances when there is strong flight motivation but the flight is blocked among both animals and human beings [9]. Based on the recent formulation of SRT, entrapment would activate the Involuntary Defeat Strategy (IDS)--a genetically hard-wired psychobiological response, deteriorate the initial sense of defeat and produce a chronic or excessive IDS response, form a “depressogenic feedback loop”, and finally contribute to the development of depression [28]. According to this demonstration, entrapment can act as an entry point to prevent depression.

For entrapment assessment, the Entrapment Scale (ES) which contains 16 item (10 external entrapment items and 6 internal entrapment items) was first proposed by Gilbert and Allan in 1998 [9] and it was suggested by Paul Gilbert that the ES should be divided into external and internal subclass [29]. The ES has been translated into Chinese by Ruijie Gong and applied in students [30], transgender sexual women [31], men who have sex with men [32] successfully. However, the validity and reliability of Chinese version of ES among MWs are still unrevealed.

What is more, although the association depression and entrapment is confirmed among patients [33], transgender women sex workers [31], and men have sex with men [32], studies among MWs are still limited. The prospective role of entrapment in depression had been proved by Griffiths, A. W, who investigated the longitudinal effects among groups recruited from the workplace and community in economical deprived areas [34]. In 2011, Manuel Trachsel tested the German Adaption of the ES and Assessing the Relation to Depression and certified the entrapment role in explaining variances in the depression [35]. Since the Chinese version of ES hasn’t been applied in MWs yet, the relationship between entrapment and depression of this group hasn’t been reported in China.

In this study, we have two aims: 1) Whether the Chinese version of ES could be applied to measure MWs entrapment effectively; 2) How is the association between depression and entrapment in MWs; 2) What is the optimal cut-off value of entrapment to predict depressive symptoms in MWs based on the applicable ES.

## Materials and methods

### Study design and data collection

According to a previous study in 2011, Shenzhen MWs’ prevalence of clinically relevant depression was 21.4% [36]. We assumed the significant level and permissible error to be 0.05 and 0.0214 respectively, as the design effect for a stratified multi-stage sampling was equal or smaller than 1, the expected sample size should be 1411.

Our MWs sample was a secondary analysis of a cross-sectional study which conducted from October to December 2019 among industrial workers in Longhua district of Shenzhen--a district contained more than 1000 industrial enterprises and one million of factory workers in 2018 [37–39]. A stratified multi-stage sampling approach was used for recruitment. Considering the ratios for workers number of different types of factories, 16 factories which contain a total of 513,215 industrial workers were randomly selected, including four mechanical processing plants, three electronic devices manufacturers, three printing and dyeing factories, two chemical raw materials plants, one smelter, one garment factory, one food and beverage manufacturer, and one other factory. Given the median number of workers in a typical workshop of factories in Longhua was about 40 to 50, a total of 59 workshops (3 or 4 workshops in each factory) were selected. Employees over 18 years old and being full-time employed from the randomly selected workshops were invited to visit Longhua District Center for Disease Control and Prevention (CDC). At the CDC, our trained fieldworkers briefed the study to the eligible 2700 workers from the selected workshops and 2023 workers completed a self-administered questionnaire. Then a total of 1805 MWs were extracted from the 2023 workers. Guarantees of anonymity, the right to quit at any time, and refusal without any consequence were made to safeguard participants’ rights. Written informed consent was obtained. We gave a cash coupon of ¥20 (US2.60) to participants who completed our survey.

### Measures

#### Design of the questionnaire

Two public health researchers, an epidemiologist, a health psychologist, a health communication expert, and a factory worker formed a panel to develop our questionnaire. And a pilot-test had been done among 20 factory workers to assess the clarity and readability of questionnaire. According to the comments of these 20 workers, the panel revised and finalized the questionnaire. These 20 workers were not included in the actual survey.

#### Psychological Variates- entrapment

We used the Chinese version of ES to assess entrapment. ES is a self-report scale designed by Paul Gilbert and Steven Allan in the 1998 to identify the subjective experiences of entrapment [9]. It has been shown to not only have well validity but also the reliability in diverse settings and multicultural populations [30, 35, 40]. And it was first professionally translated into Chinese in 2019 by Ruijie Gong, of which the validity and reliability has been proved among medicine students and the Cronbach’s for this scale in the study was 0.96 (0.94 for external and 0.93 for internal) [30].

A total of 16 items are included in the scale. Options for each item range from “not at all”, “a little bit,” “moderately,” “quite a bit,” and “extremely” correspond to scores of 0–4. The total score ranges from 0 to 64. A higher score indicates a stronger sense of entrapment. The Cronbach’s alpha was 0.902.

#### Psychological Variates-depressive symptoms

We used the validated Chinese-version of the 10 item Center for Epidemiologic Studies Depression Scale (CES-D-10) to assess the depressive symptoms of participants in the past week. Options for each item range from “rarely or none of the time” to “all of the time”, corresponding to scores of 0–3. Item 5 and 8 need to be scored inversely. Final score is the sum of the 10 graded items with scores 10 or greater considered as depressed mood [14]. The Cronbach’s alpha was 0.902.

#### Sociodemographic Variates

Basic demographic information covers age, gender, hometown type, marital status, numbers of children, education, income, years of work experience and drinking.

Drinking behavior was assessed by the World Health Organization’s Alcohol Use Disorders Identification Test (AUDIT) which consists of 10 questions, the first 8 are five-level scoring, and the last 2 are three-level scoring. Self-report scores ≥20 were regarded as with alcohol consumption problems [41].

### Data analysis

Sample’s demographic and behavioral characteristics were described as numbers and proportions by SPSS v.22. The Chi-square test and binary logistic regression proceeded in SPSS v. 22 were used to select potential factors that may influence the association between entrapment and depression. The reliability coefficients (Cronbach’s α and Spearman-Brown r) were calculated through SPSS v.22. The Exploratory Factor Analysis (EFA) consisted of Kaiser-Meyer-Olkin (KMO) test and Bartlett’s test of sphericity as well as the Confirmatory Factor Analysis (CFA) aiming to test the model fit indices and convergent validity were conducted through SPSS v. 22 and AMOS v. 24. The ratio of chi-square and degrees of freedom (X^2^/df) between 2 and 5, Root Mean Square Error of Approximation (RMSEA) below 0.08, Goodness of Fit Index (GFI) and Comparative Fit Index (CFI) greater than 0.9 indicate a good model fitness [42]. After proving the reliability and constructive validity of the Chinese ES, we ran the binary regression to analyze the relationship between entrapment and depressive symptoms. The scores of the Chinese ES were demonstrated in the form of median (Inter-Quartile Range, IQR). Finally, the Receiver Operator Characteristic (ROC) was performed by R × 64 3.6.2 for illustrating the sensitivity and specificity of ES for predicting depression.

## Results

### Sociodemographic characteristics

Sociodemographic characteristics and their relations to depression among 1805 workers are summarized in Table 1. The mean age of those workers was 31.1 years, ranging from 23.2 to 39.0 years. Of the participants, the majority (69.4%) came from rural areas of China. More than half of them (53.6%) were married, 39.8% were unmarried, and only 2.6% were divorced or widowed. Besides, 72 people refused to disclose their marital status. The numbers of children were 44.8, 49.0 and 6.2% for none, 1 or 2 children, or more than 2 children, respectively. As of the date of the survey, most of the participants (64.5%) had worked over 6 years. Among the participants, there were around 33% of participants with depressive risk.

**Table 1:** Sociodemographic characteristics (*N* = 1805)

Demographic characteristics	Total N(%)	CES-D-10 ≥ 10 N(%)
**Age group (years)**		
< 30	830 (46.0%)	300 (36.1%)
30–39	719 (39.8%)	233 (32.4%)
> 39	256 (14.2%)	73 (28.5%)
**Gender**		
Male	1214 (67.3%)	421 (34.7%)
Female	591 (32.7%)	185 (31.3%)
**Hometown**		
Urban	318 (17.6%)	95 (29.9%)
Rural	1253 (69.4%)	430 (34.3%)
Unkown	234 (13.0%)	81 (34.6%)
**Marital status**		
unmarried	719 (39.8%)	276 (38.4%)
married	967 (53.6%)	290 (30.0%)
Divorce/Widowed	47 (2.6%)	17 (36.2%)
Other or Unwilling to disclose	72 (4.0%)	23 (31.9%)
**Numbers of children**		
0	808 (44.8%)	304 (37.6%)
1 or 2	885 (49.0%)	264 (29.8%)
more than 2	112 (6.2%)	38 (33.9%)
**Education**		
Less than high school	1059 (58.7%)	342 (32.3%)
High school	585 (32.4%)	203 (34.7%)
College degree or above	113 (6.3%)	44 (38.9%)
Unknown	48 (2.7%)	17 (35.4%)
**Income (yuan per month)**		
< 3000	166 (9.2%)	64 (38.6%)
3000–4999	1099 (60.9%)	375 (34.1%)
> 4999	484 (26.8%)	145 (30.0%)
Unknown	56 (3.1%)	22 (39.3%)
**Years of work experience**		
0–2 years	346 (19.2%)	126 (36.4%)
3–5 years	295 (16.3%)	109 (36.9%)
6–10 years	612 (33.9%)	211 (34.5%)
> 10 years	552 (30.6%)	160 (29.0%)
**Drinking** ^a^ **(AUDIT scores)**		
1–7	1411 (78.2%)	457 (32.4%)
8–15	235 (13.0%)	83 (35.3%)
16–19	48 (2.7%)	12 (25.0%)
> =20	111 (6.1%)	54 (48.6%)

^a^Drinking were based on situation of the past year.

Through the analysis of binary logistic regression (Table 2), five sociodemographic variates display a significance in relation to depression. In univariate regression, compared with unmarried group, the married group was less likely to have depressive symptoms (univariate odds ratio (ORu) = 0.69, 95% Confidence Interval (CI) = 0.56–0.84); Compared with the participants without children, those owned 1 or 2 children had lower risk of depression symptoms (ORu = 0.71, 95% CI = 0.58–0.86); MWs who had been working over 10 years were less possible to get depression symptoms (ORu = 0.71, 95% CI = 0.54–0.95) when compared with those who had worked less than 2 years; For the workers with income higher than 4999 yuan per month, they had lower risks of depressive symptoms (ORu = 0.68, 95% CI = 0.47–0.99); For the low risks of alcohol use disorders group, the group with high risk of alcohol use disorders was more vulnerable to suffer depressive symptoms (ORu = 1.98, 95% CI = 1.34–2.92) than them. In the multivariable regression for demographic and behavioral variables, the income was the only factor that significantly associated with depression. The multivariate odds ratio (ORm) = 0.57, 95% CI = 0.38–0.87 when compared workers with > 4999 yuan per month with < 3000 yuan per month.

**Table 2:** Significant binary logistic regression results for sociodemographic variables

Demographic characteristics	ORu	ORm
**Marital status**		
unmarried	1	
married	0.69 (0.56,0.84) **	
Divorce/Widowed	0.91 (0.49,1.68)	
Other or Unwilling to disclose	0.75 (0.45,1.26)	
**Numbers of children**		
0	1	
1 or 2	0.71 (0.58,0.86) **	
more than 2	0.85 (0.56,1.29)	
**Income (yuan per month)**		
< 3000	1	1
3000–4999	0.83 (0.59,1.16)	0.73 (0.50,1.05)
> 4999	0.68 (0.47,0.99)*	0.57 (0.38,0.87) **
**Years of work experience**		
0–2 years	1	
3–5 years	1.02 (0.74,1.41)	
6–10 years	0.92 (0.70,1.21)	
> 10 years	0.71 (0.54,0.95) *	
**Drinking** ^a^ **(AUDIT scores)**		
1–7	1	
8–15	1.14 (0.85,1.52)	
16–19	0.70 (0.36,1.35)	
> =20	1.98 (1.34,2.92) *	

*p < 0.05;**p < 0.01;***p < 0.001

*ORu* Univariate odds ratio, *ORm* Multivariate odds ratio (In multivariate regression CES-D was dependent variable and all demographic variables were independent variables).

^a^ Drinking were based on situation of the past year.

### Constructive validity and realibility

Considering that it was the first time for the Chinese version of ES to be applied among MWs, we verified its validation before calculating its scores to analyze the association between entrapment and depression. 1805 MW were randomly divided into two groups, 902 for EFA and 903 for CFA.

#### Exploratory factor analysis

To conduct the optimal factor structure of the Chinese ES, we firstly conducted a KMO test and a Bartlett’s test to test the feasibility of applying this sample to factor analysis. The outcomes of the KMO test, which sampling adequacy was 0.961, and of the Batlett’s test (X^2^ = 11,574.461, *p* < 0.001) indicated the data was appropriate for EFA. Then, we applied a principal-axis EFA on the covariance matrix of the 16 items from the Chinese ES. The principal component extraction statistics showed that there were 2 factors with eigenvalues greater than 1 [43]. The two eigenvalues were 9.783 and 1.112 totally accounting for 68.091% of the variance in items, suggesting that the two-dimensional scale was suitable. The factor loading was acceptable as except the first item which factor loading was 0.542, other items were between 0.65 and 0.85.

#### Confirmatory factor analysis

Confirmatory factor analysis was used to compute and compare the model fit of two models, illustrated through the ratio of chi-square and degrees of freedom, root mean square error of approximation, goodness-of-fit index, and comparative fit index. The first model was the two-dimensional one according to the outcome of EFA; the second model was just a one-dimensional model. The results in Supplementary Table 1 suggested the model fit indices of both models were acceptable.

Model 1, the most acceptable one in model fit, was applied in the further confirmatory factor analysis (Supplementary Table 2). Each parameter was significant. As all of the squared multiple correlations (SMC) were all greater than 0.35 [44], acceptable item reliabilities were existed. All composite reliability (CR) were bigger than 0.6 [44], revealing the good internal consistency. The good convergency validity of each model was proved by average of variance extracted (AVE), which were greater than 0.5 [44].

#### Reliability

The Cronbach’s α and Spearman-Brown coefficient for the Chinese ES were 0.956 (Model 1: 0.935 for external entrapment, 0.917 for internal entrapment; Model 2:0.941 for external entrapment, 0.916 for internal entrapment) and 0.917 respectively, indicating the good internal consistency reliability and split-half reliability.

### The relationship between entrapment and depression

Table 3 depicted the psychological health conditions of entrapment among the participating workers. The binary logistic regression displayed a significant positive relationship between depression and entrapment, both the internal and external entrapment. After adjusted by sociodemographic factors (Marital status, Numbers of children, Income, Years of work experience and Drinking) which were significant in above binary logistic regression, such significant correlation still existed.

**Table 3:** MWs’ entrapment conditions and relationship between entrapment and depression. (*N* = 1805)

Variates		Median (IQR)	ORu(95% CI)	ORa(95% CI)
Entrapment	CSE-D-10 < 10	3 (10)	1.13 (1.11,1.14) ***	1.13 (1.11,1.15) ***
	CSE-D-10 ≥ 10	20 (21)		
IE^a^	CSE-D-10 < 10	0 (2)	1.30 (1.26,1.36) ***	1.30 (1.26,1.34) ***
	CSE-D-10 ≥ 10	5 (8)		
EE^a^	CSE-D-10 < 10	1 (5)	1.19 (1.17,1.22) ***	1.20 (1.17,1.22) ***
	CSE-D-10 ≥ 10	11 (14.25)		

*p < 0.05;**p < 0.01;***p < 0.001

^a^ IE and EE are sub-dimensioned based on the model 1, the two dimensional model

*IE* Internal Entrapment, *EE* External Entrapment, *IQR* Inter-Quartile Range, *ORu* Univariate odds ratio, *CI* Confidence Interval, *ORa* Odds ratio obtained from forward stepwise multivariate logistic regression adjusted by” Marital status”; “Numbers of children”; “Income “; “Years of work experience”, and “Drinking”

### Sensitivity and specificity of the ES for predicting depressive symptoms

**Fig. 1:**
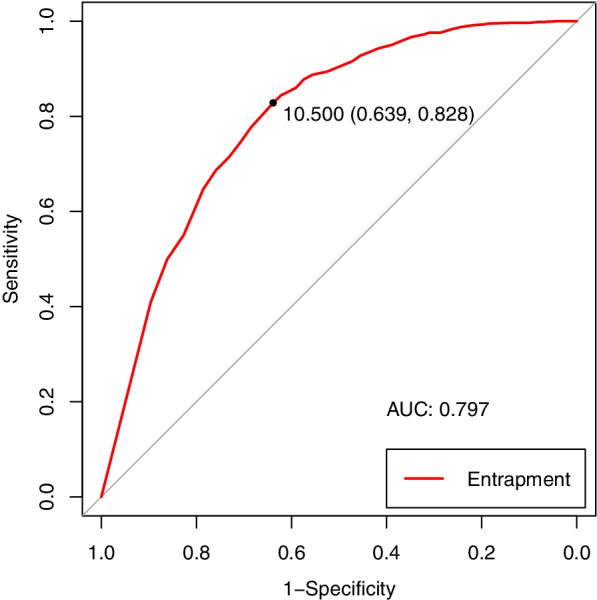
ROC curve for ES to predict depressive symptoms

The ROC was applied for Chinese ES to predict the existence depressive symptoms. It suggested that the Chinese ES can predict depression diagnosed by CES-D-10 among workers well (area under the ROC curve was 0.797, 95%CI = 0.774,0.820). 11 was the optimal cut-off value for predicting depression with sensitivity of 63.9% and specificity of 82.8% (Fig. 1).

## Discussion

This study confirmed the relationship between entrapment and depressive symptoms narrated by SRT. According to the result of constructive analysis, the two-dimensional ES is preferable for MWs, which was also recommended by the original study [9]. However, item 11(“I would like to get away from who I am and start again”) which was used to belonging to the IE in the original study was classified in EE in this study, indicating a possible different classification way in MWs when applying ES. Considering the studies on the constructure of ES were still inconclusive [30, 35, 45], we applied both the two-dimensional constructure constructed by this study and the one-dimensional constructure for binary logistic regression. We found that Entrapment and its subscales are all significantly related to depressive symptoms. Combining the above result with ROC curve, among MWs, the scores of ES over 11 may indicate the possibility of the existence of depressive symptoms.

Our study suggested that MWs with less family supports and less working experience were more likely to suffer depressive symptoms. This is parallel to the prior research on Shenzhen MWs [36]. It is common for MWs to experience work-family conflict [46]. The family support, such as family belongings has been proved to be effective in decreasing the association between entrapment and PTSD [47], as well as suicide [48]. Previous study suggested a U-shaped mental health status as time of migration passed by [36], while our study found the protective effect of the longest duration of working experience-- the MWs with over 10 years working experience were less likely to obtain depressive symptoms. There is a possibility that the social support from working environment may compensate the deficiency of family supports [46]. Thus, we emphasize the importance of family support in reducing entrapment and preventing depression. Considering the family-work conflict, increasing support at work may compensate the loss of family support.

Our result exemplified the low socioeconomic status of MWs. The education levels of more than 90% of participants are lower the high school or technical secondary school level; around 70% of participants earns 3000–4999 yuan per month (Table 1)**,** being lower than the local average level (70,233 yuan per year for urban private company or organization and 125,612 yuan/ year for urban non-private Urban non-private company or organization) [49]. After adding all demographic and behavioral factors into consideration, the income became the only significant factor associated with depressive symptom, indicating participants with less income had a higher risk of developing depressive symptoms (Table 2). The economic factor role in eliciting depression might be related to the social income inequality as Lin’s study in 2017 revealed that MWs with large income inequality were worst in health statues and had worse social integration [11]..

There are overt limitations in this study. Firstly, self-report scales own inevitable information bias, and we applied the scale based on the participants self-report to assess the depressive symptoms which was comparatively less accurate than clinical interviews. And as we kept the number of participants from each factory to be similar during the sampling, it might affect the representative of our sample since the factory size could be different in reality. What’s more, as our participants are Chinese workers who are migrant, how broadly and confidently the conclusion becomes accepted in other regions or non-MWs are still under consideration. Besides, as this was a cross-sectional study with self-report questionnaire, we cannot further explore the causal sequence between entrapment and depressive symptoms and analyze whether there is an optimal cut-off point for ES to foreseen depression ahead of time instead of the current depressive symptoms.

In view of the mutual interaction between entrapment and depression as well as the multiple mental disorders associated with entrapment, not only the depression [35], more researches are needed for evaluating the effects of ES in early detection of depression.

## Conclusions

The present study first proved the validity and reliability of Chinese version ES among Chinese MWs and revealed a positive relationship between entrapment and depression among MWs, suggesting 11 was the optimal cut-off point for applying the ES to predict depressive symptoms among MWs. It indicated the future applicable value of the ES in clinical research and practice and the need of future studies of prospective research on the entrapment role of predicting depression.

## Supplementary Information

**Additional file 1.**

## Data Availability

The data used in the current study are available from the corresponding author on reasonable request.

## Abbreviations

AUDIT: Alcohol Use Disorders Identification Test
AVE: Average of Variance Extracted
CDC: Center for Disease Control and Prevention
CES-D-10: 10 item Center for Epidemiologic Studies Depression Scale
CFA: Confirmatory Factor Analysis
CFI: Comparative Fit Index
CI: Confidence Interval
CR: Composite Reliability
df: Degrees of freedom
EE: External Entrapment
ES: Entrapment Scale
EFA: Exploratory Factor Analysis
GFI: Goodness of Fit Index
IDS: Involuntary Defeat Strategy
IE: Internal Entrapment
IQR: Inter-Quartile Range
KMO: Kaiser-Meyer-Olkin
MWs: Migrant Workers
ORa: Adjusted odds ratio
ORm: Multivariate odds ratio
ORu: Univariate odds ratio
RMSEA: Root Mean Square Error of Approximation
ROC: Receiver Operator Characteristic
S.E.: Standard errors of Estimates
SMC: Squared Multiple Correlations
SRT: Social Rank Theory
Std.: Standardized estimates
X^2^: Chi-square
Unstd.: Unstandardized estimates
