# Supplementary material for: The association between entrapment and depression among migrant workers in China: a social rank theory based study
Source: BMC Psychiatry. 2022 Jan 6;22:17. doi: 10.1186/s12888-021-03665-6 (PMC8740036; doi:10.1186/s12888-021-03665-6)
Supplement: Supplementary file 1 — Additional file 1. [file 12888_2021_3665_MOESM1_ESM.docx]

Supplementary Tables

**Supplementary Table 1.** Model fit indices (n = 903).

| **Model index** | **X²** | **df** | **X² /df** | **RMSEA** | **GFI** | **CFI** |
| --- | --- | --- | --- | --- | --- | --- |
| Model 1 | 818.066 | 103 | 7.942 | 0.083 | 0.896 | 0.944 |
| Model 2 | 1491.004 | 104 | 14.337 | 0.122 | 0.786 | 0.872 |

Model 1: two-dimensional model; Model 2: one-dimensional model; X²: chi-square; df: degrees of freedom; RMSEA: root mean square error of approximation; GFI: goodness-of-fit index; CFI: comparative fit index.

**Supplementary Table 2.** Reliability and convergent validity table of Model 1 (n = 903).

|  | **Item** | **Parameter significance estimation** | | | | **Convergent validity** | | | |
| --- | --- | --- | --- | --- | --- | --- | --- | --- | --- |
|  | **No.** | **Unstd.** | **S.E.** | **T value** | **P** | **Std.** | **SMC** | **CR** | **AVE** |
| EE^†^ | 1 | 1 |  |  |  | 0.683 | 0.466 | 0.534 | 0.945 |
|  | 2 | 1.239 | 0.050 | 24.550 | *** | 0.828 | 0.686 | 0.314 |  |
|  | 3 | 1.055 | 0.046 | 22.765 | *** | 0.768 | 0.59 | 0.41 |  |
|  | 4 | 1.175 | 0.048 | 24.466 | *** | 0.832 | 0.692 | 0.308 |  |
|  | 5 | 1.346 | 0.056 | 23.984 | *** | 0.808 | 0.653 | 0.347 |  |
|  | 6 | 1.229 | 0.052 | 23.535 | *** | 0.795 | 0.632 | 0.368 |  |
|  | 7 | 1.389 | 0.059 | 23.507 | *** | 0.791 | 0.626 | 0.374 |  |
|  | 8 | 1.046 | 0.048 | 21.876 | *** | 0.735 | 0.54 | 0.46 |  |
|  | 9 | 1.197 | 0.053 | 22.759 | *** | 0.767 | 0.588 | 0.412 |  |
|  | 10 | 1.032 | 0.045 | 22.737 | *** | 0.768 | 0.59 | 0.41 |  |
|  | 11 | 1.106 | 0.047 | 23.407 | *** | 0.795 | 0.632 | 0.368 |  |
| IE^†^ | 12 | 1 |  |  |  | 0.759 | 0.576 | 0.424 | 0.918 |
|  | 13 | 1.145 | 0.038 | 30.108 | *** | 0.877 | 0.769 | 0.231 |  |
|  | 14 | 1.083 | 0.038 | 28.427 | *** | 0.844 | 0.712 | 0.288 |  |
|  | 15 | 1.197 | 0.042 | 28.325 | *** | 0.843 | 0.711 | 0.289 |  |
|  | 16 | 1.136 | 0.041 | 27.684 | *** | 0.831 | 0.691 | 0.309 |  |

*p<0.05;**p<0.01;***p<0.001;

^†^: IE and EE are sub-dimensioned based on the model 1, the two-dimensional model;

Unstd.: Unstandardized estimates; S.E.: Standard errors of Estimates; Std.: Standardized estimates; SMC: Squared Multiple Correlations; CR: Composite Reliability; AVE: Average of Variance Extracted.
